# Pregnancy Outcomes Among Teenagers at a National Referral Hospital in Uganda

**DOI:** 10.1155/2024/6975966

**Published:** 2024-06-11

**Authors:** Mike Nantamu Kagawa, Otto Alex Owori, Miriam Nakalembe

**Affiliations:** ^1^ Department of Obstetrics & Gynecology Makerere University College of Health Sciences, Kampala, Uganda; ^2^ Department of Obstetrics & Gynecology Paragon Hospital Bugolobi, Kampala, Uganda

**Keywords:** adverse outcomes, fetal outcome, maternal outcome, teenage pregnancy

## Abstract

**Introduction:** Teenage pregnancy is a global public health challenge, and it is a major contributor to the high maternal and neonatal morbidity and mortality rates reported in sub-Saharan Africa and Uganda. However, there is a paucity of data regarding pregnancy outcomes and their associated factors among teenagers in Uganda. The purpose of this study was to determine the prevalence and factors associated with pregnancy outcomes among teenagers who delivered at a National Referral Hospital in Kampala, Uganda.

**Materials and Methods:** This cross-sectional study was conducted among teenage mothers who delivered at a National Referral Hospital in Kampala, Uganda. Consecutive participant recruitment was done for those who fulfilled the eligibility criteria. The outcomes of interest included adverse maternal outcome with obstructed labor being used as a proxy and adverse fetal outcomes with birth asphyxia used as a proxy. Logistic regression analysis was used to determine the association between independent and dependent variables with a 5% level of statistical significance (*α* = 0.05).

**Results:** Teenage pregnancy was associated with adverse maternal outcomes which included obstructed labor (18%) and preterm labor (5.5%). There were no maternal deaths during the study period. Adverse fetal outcomes observed in this study population included low birth weight (83%), birth asphyxia (18%), and stillbirth (4%). The only factor associated with adverse maternal outcome was gestational age where teenage mothers had 4 times likelihood of delivering before 37 weeks. Relatedly, teenage mothers had an 81% chance of having a preterm birth.

**Conclusion:** Teenage pregnancy was generally not associated with adverse maternal or fetal outcomes except for preterm birth. The reasons for adverse pregnancy outcomes may reflect a combination of gynecological and biological immaturity, as well as adverse socioeconomic pressures.

## 1. Introduction

Teenage pregnancy remains a global public health challenge, with an estimated 21 million girls aged 15–19 years in developing countries becoming pregnant, 12 million of whom give birth every year [1].

Sub-Saharan Africa had the highest prevalence of teenage pregnancy in the world in 2013 [2] with births to teenage mothers accounting for more than half of all the births [3]. Uganda has one of the highest rates of teenage pregnancy in the world at 25% which is more than twice the global estimate of 11% of all births [4].

Pregnant teenagers are at a higher risk of adverse maternal conditions, including preterm labor, puerperal sepsis, postpartum hemorrhage, and maternal trauma, and a high rate of cesarean sections for cephalopelvic disproportion, fetal distress, and preterm births. This same population of teenage mothers is at high risk of adverse fetal outcomes such as stillbirths, birth asphyxia, respiratory distress syndrome, admission to NICU, and early neonatal death (ENND) [5–8].

Several studies in developed and developing countries have shown that most teenage pregnant mothers do not attend the required antenatal care (ANC) visits or present late in labor, which has been associated with poor obstetric and fetal outcomes. Most of these studies were based on retrospective data collection, making them prone to missing data. No study has examined teenage mothers in Uganda, and the prevalence and factors associated with adverse maternal and fetal outcomes remain largely unknown. Therefore, this study is aimed at determining the prevalence of, and factors associated with adverse maternal and fetal outcomes among teenagers who delivered at a National Referral Hospital in Uganda.

## 2. Materials and Methods

This was a cross-sectional study.

The study was carried out in the postnatal wards of Mulago National Referral Hospital, which also functions as a teaching hospital for Makerere University College of Health Sciences. At the time of the study, the Department of Obstetrics and Gynecology was located at Kawempe Hospital, which had 27,202 deliveries in 2017 (1 January–31 December, departmental records, unpublished data).

### 2.1. Study Population

The study population included teenagers aged between 13 and 19 years who delivered at the Kawempe Hospital. All postnatal women aged 13–19 years admitted to postnatal wards within 24 hours postdelivery who consented to the study were included in the study. Women aged 13–19 years who delivered before arrival at the hospital were excluded from the study.

### 2.2. Sample Size Estimation

To determine the prevalence of adverse maternal outcomes, the sample size was determined by the Kish Leslie formula, using preterm delivery as the variable of interest, based on a study conducted by Kumar et al. [9] which gave a sample size of 296. For the prevalence of adverse fetal outcomes, the Cochran formula was used with the assumption that the study would be completed in a 2-month period and there were approximately 250 teenage deliveries in Mulago Hospital per month. This resulted in a final sample size of 217 patients. The Fleiss formula for comparing two proportions [10] was used to calculate the sample size for the factors associated with adverse maternal outcomes. Based on the study by Egbe et al. [11], the sample size was 186 teenage women. For factors associated with adverse fetal outcomes, the calculation was based on the study by Bayo et al. and gave a sample size of 330 teenage women [12]. The largest sample size of 330 was used, which was increased by a factor of 10% to cater for incomplete questionnaires and other errors in data collection. The sample size of this study was 363 teenagers.

## 3. Results and Discussion

The median age of the participants was 18 years, and 76% of the participants were older than 18 years. About 5% had no formal education, 61% had studied to the primary school level, and 34% had attained secondary school status. Approximately 2% of the participants were underweight and 6% were obese (Table 1).

The majority of the teenagers did not have any adverse maternal or fetal outcomes. There were 17 stillbirths with seven fresh stillbirths (FSBs), seven macerated stillbirth (MSB), and three ENND (Table 2).

The only factor associated with adverse maternal outcome was gestational age where teenage mothers had 4 times likelihood of delivering before 37 weeks (Table 3).

There were no factors found to be statistically significant in association with adverse fetal outcome except gestational age (Table 4). Teenage mothers had an 81% chance of delivery before 37 weeks.

## 4. Discussion

The median age of the participants was 18 years (IQR = 5.0), with approximately 76% of the teenage mothers between 18 and 19 years. Approximately 75% of the teenagers were unemployed (Table 1). This agrees with results of the UDHS 2016 which indicated that the teenagers in the lower quintile of wealth tend to begin childbearing earlier than those in the highest quintile of wealth, 34% versus 15%, respectively [4]. Of all the study participants, 34% had achieved secondary school education and above, with 61% dropping out at primary school level and 5% not having received any formal education (Table 1). This is similar to findings of the USAID-YALI (Young African Leaders Initiative) report of 2018 which reported a remarkable drop in the number of adolescent girls who continue their education beyond primary school; while 87% of girls enroll in primary school, only 39% complete secondary school. It is also worth noting that one of the government's strategies for preventing teenage pregnancies is to keep girls in school. Therefore, our findings reinforce the government's initiative to keep girls in school to minimize the risk of teenage pregnancy.

The prevalence of obstructed labor among teenagers was 18%, whereas 5% had preterm labor. No maternal death occurred during the study period (Table 2). This contrasts with a hospital study conducted in India, which reported a higher prevalence of preterm labor (27.45%) among teenage pregnant mothers [13]. This could be attributed to the fact that these studies were carried out on different races in totally different environments. Most of the babies delivered were below 2.5 kg. This is similar to a study done in Oman where teenage mother had a higher prevalence of low birthweight babies [14]. The low birthweight babies in this particular group may be associated with low ANC attendance and poor maternal nutrition which may be prevalent among teenage mothers bearing in mind the fact that many teenage mothers may be of low socioeconomic status [15]. The prevalence of birth asphyxia was 18% (Table 2). This is similar to a study done in Mogadishu, Somalia, which reported a prevalence of 13.3%. This may be because teenage mothers may be more likely to have a contracted pelvis because of their young age which may predispose them to obstructed labor and caesarian section. The prevalence of stillbirths (FSB, MSB, and ENNDs combined) was 4.4% (Table 2). While this may appear low, it fits in with reports that indicate that teenage mothers are prone to stillbirths because of the increased incidence of contracted pelvis, preterm labor, and caesarian section [16].

The only factor associated with adverse maternal outcomes among teenage mothers was preterm labor (delivery before 37 weeks). Teenage mothers were 4 times more likely to deliver before 37 weeks than at 37 weeks and above (AOR = 3.78, 95% CI 1.67–8.55) (Table 3). This is similar to a study done in Sao Luis, Northern Brazil, which showed that teenage mothers were prone to preterm labor (OR = 1.70, 95% CI 1.02–3.08) [14]. Some studies have however reported no association even after controlling for confounders such as socioeconomic status [17].

In this study, preterm birth was the only adverse fetal factor associated with teenage pregnancy. Teenage mothers had an 81% chance of having a preterm birth (birth before 37 weeks) (Table 4). All the other factors studied such as maternal age, education level, marital status, religion, employment status, household income, maternal BMI, and partograph use during labor did not show any significant association. This is similar to a study conducted in Cameroon, which showed no association between adolescent pregnancy and stillbirths and intrauterine growth retardation but demonstrated an association between adolescent pregnancy and low birth weight, prematurity, and ENND [18]. Another study conducted in Pakistan also indicated no association between teenage pregnancy and adverse neonatal outcomes, especially in a setting of good ANC [19]. Studies investigating the association between fetal outcome and maternal age have yielded contrasting results. Adverse fetal outcomes in teenagers have been postulated to be due to both biological immaturity and socioeconomic factors. Teenagers are more prone to poor nutrition, emotional stress, illicit drug use, and inadequate ANC, and the authors have postulated that improved socioeconomic status of teenagers can lead to better pregnancy outcomes [17]. While poor socioeconomic status is associated with adverse pregnancy outcome, it has also been reported to be a driver for high teenage pregnancy rates among teenagers who get involved in early sexual encounters to make a living, which makes it a viscous cycle [20]. The difference between our findings and other studies may be because of differences in the socioeconomic background of the teenagers included in the study.

## 5. Conclusions

Teenage pregnancy was generally not associated with adverse maternal or fetal outcomes except for preterm birth. The reasons for adverse pregnancy may reflect a combination of gynecological and biological immaturity, as well as adverse socioeconomic pressures. The interpretation of these results may be limited in relation to the bigger picture of adverse maternal and fetal outcomes, and a study comparing adverse outcomes between teenage mothers and mothers above 19 years may provide better results.

## Figures and Tables

**Table 1:** Demographic characteristics and physical examination of participants, *N* = 363.

**Variable**	**Number (%)**
*Maternal age (median 18.0,IQR* = 5.0)	
Below 18 years	87 (24.0)
18 and above	276 (76.0)
*Education level*	
None	19 (5.2)
Primary school	221 (60.9)
Secondary school and above	123 (33.9)
*Employment status*	
Employed	90 (24.8)
Unemployed	273 (75.2)
*Household monthly income*	
< 100,000	48 (13.2)
100,000–300,000	166 (45.7)
> 300,000	149 (41.1)
*Marital status*	
Married^∗^	227 (62.5)
Not married	136 (37.5)
*BMI (median 24.56,IQR* = 16.64)	
Underweight (less than 18.5)	7 (2.0)
Normal weight (18.5–24.9)	206 (56.8)
Overweight (25–29.9)	127 (34.1)
Obese (30 and above)	23 (6.3)

^∗^This group included married and living together in a union even if not officially married.

**Table 2:** Adverse maternal and fetal outcomes of study participants, *N* = 363.

	**Number (%)**
*Maternal outcomes*	
Obstructed labor	
Yes	65 (17.9)
No	298 (82.1)
Preterm labor	
Yes	20 (5.5)
No	343 (94.5)
Maternal death	
Yes	0 (0.0)
No	363 (100)
*Fetal outcomes*	
Birth weight	
2.5 kg and above	63 (17.3)
Below 2.5 kg	300 (82.6)
Birth asphyxia	
Yes	63 (18.0)
No	286 (82.0)
Birth outcome	
Live birth	346 (95.6)
Stillbirths	17 (4.4)
FSB	7
MSB	7
ENND	3

**Table 3:** Factors associated with adverse maternal outcomes.

**Characteristic**	**Crude OR (95% CI)**	**P** **value**	**Adjusted OR (95% CI)**	**P** **value**
*Maternal age*				
Per year increase	1.06 (0.88; 1.29)	0.526	1.02 (0.82; 1.27)	0.850
*Education level*				
None	1		1	
Primary	2.34 (0.75; 7.30)	0.281	2.49 (0.72; 8.58)	0.350
≥ Secondary	2.57 (0.81; 8.19)		2.50 (0.68; 9.14)	
*Marital status*				
Married	1		Base	0.239
Not married	0.66 (0.43; 1.04)	0.72	0.73 (0.44; 1.23)	
*Religion*				
Catholic	1		1	
Protestant	1.15 (0.64; 2.08)	0.609	1.37 (0.72; 2.61)	0.473
Moslem	0.91 (0.52; 1.60)		0.87 (0.49; 1.62)	
Others	0.75 (0.41; 1.38)		0.83 (0.42; 1.64)	
*Employment status*				
Employed	1		1	
Unemployed	1.33 (0.81; 2.19)	0.265	1.54 (0.89; 2.65)	0.122
*Household income*				
< 100,000	1		1	
100,000–300,000	0.95 (0.49; 1.87)	0218	1.02 (0.47; 2.21)	0.296
Above 300,000	1.41 (0.72; 2.77)		1.50 (0.68; 3.29)	
*Body mass index*				
Underweight	1		1	
Normal weight	1.31 (0.29; 6.01)		1.30 (0.27; 6.32)	
Overweight	1.25 (0.80; 1.97)	0.771	1.33 (0.81; 2.18)	0.682
Obese	0.93 (0.38; 2.30)		0.89 (0.32; 2.47)	
*Gestation age*				
37 and above	1		1	
Below 37 weeks	3.32 (1.59; 6.95)	0.001	3.78 (1.67; 8.55)	**0.001**
*Partograph use*				
Yes	Base		Base	
No	0.82 (0.18; 3.74)	0.802	0.79 (0.16; 3.94)	0.777

*Note:* This is the variable that showed statistical significance at multivariate analysis with a *P* value less than 0.05.

**Table 4:** Factors associated with adverse fetal outcome.

**Characteristic**	**Crude OR (95% CI)**	**P** **value**	**Adjusted OR (95% CI)**	**P** **value**
*Maternal age*				
Per year increase	0.88 (0.72; 1.08)	0.214	0.89 (0.70; 1.14)	0.350
*Education level*				
None	1		1	
Primary	1.11 (0.40; 3.04)	0.394	1.17 (0.37; 3.66)	0.741
≥ Secondary	0.80 (0.28; 2.25)		0.95 (0.28; 3.20)	
*Marital status*				
Married	1		1	
Not married	1.14 (0.72; 1.81)	0.567	1.04 (0.60; 1.81)	0.879
*Religion*				
Catholic	1		1	
Protestant	0.71 (0.38; 1.31)	0.452	0.45 (0.22; 0.90)	0.089
Moslem	1.15 (0.63; 2.11)		0.97 (0.49; 1.94)	
Others	0.83 (0.45; 1.55)		0.76 (0.37; 1.57)	
*Employment status*				
Employed	1	0.328	1	0.390
Unemployed	0.77 (0.45; 1.30)		0.77 (0.43; 1.39)	
*Household income*				
< 100,000	1	0.654	1	0.437
100,000–300,000	1.19 (0.60; 2.37)		1.04 (0.46; 2.36)	
Above 300,000	0.96 (0.48; 1.91)		0.74 (0.32; 1.69)	
*Body mass index*				
Underweight	1		1	
Normal weight	2.52 (0.30; 21.41)	0.461	2.91 (0.32; 26.72)	0.397
Overweight	0.74 (0.46; 1.18)		0.72 (0.42; 1.22)	
Obese	0.79 (0.32; 1.96)		0.65 (0.23; 1.84)	
*Gestation age*				
37 and above	1		1	
Below 37 weeks	0.19 (0.09; 0.40)	< 0.001	0.19 (0.08; 0.43)	**< 0.001**
*Partograph use*				
Yes	1		1	
No	1.61 (0.36; 7.33)	0.536	2.02 (0.40; 10.13)	0.395

*Note:* This is the variable that showed statistical significance at multivariate analysis with a *P* value less than 0.05.

## Data Availability

All data regarding this manuscript are available from the corresponding author.
